# All1371 is a polyphosphate-dependent glucokinase in *Anabaena* sp. PCC 7120

**DOI:** 10.1099/mic.0.081836-0

**Published:** 2014-12

**Authors:** Friederike Klemke, Gabriele Beyer, Linda Sawade, Ali Saitov, Thomas Korte, Iris Maldener, Wolfgang Lockau, Dennis J. Nürnberg, Thomas Volkmer

**Affiliations:** 1 Plant Biochemistry, Humboldt-Universität zu Berlin, Berlin, Germany; 2 Molecular Biophysics, Humboldt-Universität zu Berlin, Berlin, Germany; 3 Institute of Microbiology and Infection Medicine/Organismic Interactions, University of Tübingen, Tübingen, Germany; 4 School of Biological and Chemical Sciences, Queen Mary University of London, London, UK

## Abstract

The polyphosphate glucokinases can phosphorylate glucose to glucose 6-phosphate using polyphosphate as the substrate. ORF *all1371* encodes a putative polyphosphate glucokinase in the filamentous heterocyst-forming cyanobacterium *Anabaena* sp. PCC 7120. Here, ORF *all1371* was heterologously expressed in *Escherichia coli*, and its purified product was characterized. Enzyme activity assays revealed that All1371 is an active polyphosphate glucokinase that can phosphorylate both glucose and mannose in the presence of divalent cations *in vitro*. Unlike many other polyphosphate glucokinases, for which nucleoside triphosphates (e.g. ATP or GTP) act as phosphoryl group donors, All1371 required polyphosphate to confer its enzymic activity. The enzymic reaction catalysed by All1371 followed classical Michaelis–Menten kinetics, with *k*_cat_ = 48.2 s^−1^ at pH 7.5 and 28 °C and *K*_M_ = 1.76 µM and 0.118 mM for polyphosphate and glucose, respectively. Its reaction mechanism was identified as a particular multi-substrate mechanism called the ‘bi-bi ping-pong mechanism’. Bioinformatic analyses revealed numerous polyphosphate-dependent glucokinases in heterocyst-forming cyanobacteria. Viability of an *Anabaena* sp. PCC 7120 mutant strain lacking *all1371* was impaired under nitrogen-fixing conditions. GFP promoter studies indicate expression of *all1371* under combined nitrogen deprivation. All1371 might play a substantial role in *Anabaena* sp. PCC 7120 under these conditions.

## Introduction

Inorganic polyphosphate, which is a linear polymer of 10–1000 orthophosphates linked by phosphoanhydride bonds, has been found in all representative living cells, including bacteria, fungi, plants, animals and archaea (Achbergerová & Nahálka, 2011; Rao *et al.*, 2009; Remonsellez *et al.*, 2006; Scherer & Bochem, 1983). Polyphosphate is stored in the cytoplasm, where it can be visualized as metachromic inclusions (Meyer, 1902) or electron-dense granules (Jensen, 1968). Evolutionarily, polyphosphate stands as one of the earliest polymers produced in cells. Polyphosphate is considered to be the ancestor of ATP as an energy source (Resnick & Zehnder, 2000), given that hydrolysis of the phosphoanhydride bond between each orthophosphate yields free energy comparable to that generated by cleavage of ATP. In a bacterial cell, polyphosphate functions mainly as a dynamic storage compound for phosphate and energy (Harold, 1966; Kornberg *et al.*, 1956). However, many other functions have been proposed for the polymer, including those in stress responses, complexation of heavy metals, biofilm formation and virulence (Kornberg, 1995; Rashid & Kornberg, 2000; Rashid *et al.*, 2000; Tsutsumi *et al.*, 2000). In bacteria, polyphosphate metabolism is driven by two kinds of enzymes: kinases and phosphatases. Polyphosphate is synthesized by polyphosphate kinase type 1 (Ahn & Kornberg, 1990; Kornberg *et al.*, 1956), which catalyses the formation of the phosphoanhydride bonds between the growing polymer and the γ-phosphoryl residues of ATP or another nucleotide triphosphate. Conversely, polyphosphate is degraded mainly by exopolyphosphatases (Akiyama *et al.*, 1993; Kornberg *et al.*, 1999) and endopolyphosphatases (Lichko *et al.*, 2010).

Polyphosphate glucokinase (PPGK; EC 2.7.1.63), a paralogue of the ATP-dependent glucokinase (Hsieh *et al.*, 1993), catalyses the transfer of the terminal phosphoryl residue of polyphosphate to glucose in order to generate glucose 6-phosphate. The first ATP/polyphosphate-dependent glucokinase was discovered in *Mycobacterium phlei* (Szymona & Ostrowski, 1964). Since then, such enzymes have been found in many non-eukaryotic organisms (Liao *et al*., 2012; Lindner *et al.*, 2010; Pepin & Wood, 1986; Phillips *et al.*, 1993; Szymona & Widomski, 1974; Tanaka *et al.*, 2003). All but one of the known PPGKs are bifunctional, in that they are able to utilize both ATP and polyphosphate as phosphoryl donors. The sole known exception to that is the PPGK of the polyphosphate-accumulating bacterium *Microlunatus phosphovorus* (Tanaka *et al.*, 2003), which is a strictly polyphosphate-dependent enzyme.

The potential role of PPGKs in the complex metabolism of cyanobacteria has not yet been investigated. Cyanobacteria are a widespread group of oxygenic photosynthetic prokaryotes. During photosynthesis, energy is transiently stored in the energy-rich phosphoanhydride bonds of ATP molecules. Several genera of cyanobacteria perform both photosynthesis and N_2_ fixation; however, these two physiological processes are incompatible, because the oxygen-sensitive nitrogenase complex (Hill *et al.*, 1981) is the key enzyme in N_2_ fixation. The diazotrophic cyanobacteria have developed special mechanisms to allow N_2_ fixation to take place under aerobic conditions (Berman-Frank *et al.*, 2003). Some filamentous cyanobacteria, such as *Anabaena* sp. PCC 7120 (also called *Nostoc* sp. PCC 7120; hereafter *Anabaena*), form highly specialized cells called ‘heterocysts’, which fix N_2_ in a micro-oxic environment (Adams *et al*., 1981). The heterocysts are semi-regularly distributed along the filaments and rely on vegetative cells to supply them with photosynthetic products. In return, the heterocysts provide the filament with reduced nitrogen compounds (Flores & Herrero, 2010; Maldener & Muro-Pastor, 2010). In contrast, some unicellular diazotrophic cyanobacteria use a diurnal rhythm to separate N_2_ fixation and photosynthesis, protecting the nitrogenase from oxygen by employing it in the dark, when photosynthesis is quiescent (Mitsui *et al*., 1986; Toepel *et al.*, 2008).

The purpose of this study was to characterize All1371, the PPGK from *Anabaena*, *in vitro* and to explore its biological function *in vivo*.

## Methods

### 

#### Sequence analysis.

A blastp search (Altschul *et al.*, 1997) was performed against all cyanobacterial sequences available from the Integrated Microbial Genomes database (Markowitz *et al.*, 2012) and against the sequences of Section V cyanobacteria identified by Dagan *et al*. (2013). The amino acid sequence of the PPGK from *Anabaena* (*all1371*: 637231738, gene ID Integrated Microbial Genomes database) was used as query. Similar amino acid sequences of proteins with known 3D structures were identified using the structure database PDBsum (http://www.ebi.ac.uk/pdbsum/). Sequences were aligned using clustalw2 (Larkin *et al.*, 2007), and formatted with ESPript (Gouet *et al.*, 1999). Sequence similarities were determined using the emboss needle software (http://www.ebi.ac.uk/Tools/psa/emboss_needle/).

#### Bacterial strains and culture conditions.

*Anabaena* was grown in fourfold diluted medium of Allen & Arnon (1955) (AA/4 medium) with or without 10 mM KNO_3_. Liquid cultures of *Anabaena* were grown under permanent illumination with white light of 70 µmol photons m^−2^ s^−1^ at 30 °C. Cultures were grown in air lift flasks (Ø 6 cm), bubbled with air enriched with 2 % (v/v) CO_2_. Mutants were grown in the presence of 50 µg neomycin ml^−1^ or 4 µg spectinomycin ml^−1^ and 1 µg streptomycin ml^−1^. *Synechocystis* sp. PCC 6803 (hereafter *Synechocystis*) was grown on BG11 agar plates (Rippka *et al.*, 1979) additionally containing 20 mM HEPES. Liquid cultures were grown at 28 °C and under continuous illumination as described above. Liquid cultures of *Mastigocladus laminosus* SAG 4.84 and *Fischerella muscicola* PCC 7414 were grown in Castenholz medium D (8.24 mM NaNO_3_, 0.99 mM KNO_3_) or medium ND (without nitrate) (Castenholz, 1988) at 42 °C and under continuous illumination of 100 µmol photons m^−2^ s^−1^.

Chlorophyll *a* content was determined as described by de Marsac & Houmard (1988). For nitrogen starvation, exponentially grown cultures were harvested by centrifugation, washed twice with nitrate-free medium and resuspended to a final concentration of 7 µg chlorophyll ml^−1^ for further growth. *Escherichia coli* strains DH5α and BL21 (DE3) (Novagen; Merck Chemicals) were grown at 37 °C as batch culture in Erlenmeyer flasks with shaking at 300 r.p.m. in Luria–Bertani (LB) medium (Bertani, 1951) supplemented with 10 µg ampicillin ml^−1^, 150 µg neomycin ml^−1^ or 50 µg spectinomycin ml^−1^ when appropriate.

#### Construction of expression plasmid.

The *all1371* gene was amplified by PCR using genomic *Anabaena* DNA as template and oligonucleotides 5′-A**GGATCC**TACTCAATGGTGGAAGATAACGG-3′ and 5′-**GCGGCCGC**TTCTATAGTGTTTTTTCATCTC-3′ (*Bam*HI and *Not*I restriction sides highlighted in bold, stop codon underlined). The PCR product was ligated into the cloning vector pJET1.2 (Thermo Scientific) to ensure efficient restriction digests. After restriction digest of the resulting pJET-*all1371* by *Bam*HI and *Not*I, *all1371* was inserted into the vector pGEX-6P-1 (GE Healthcare), leading to pGEX_*all1371*. The manipulations were checked by restriction analysis and DNA sequencing.

#### Protein expression and purification.

*E. coli* BL21(DE3) cells were transformed with pGEX_*all1371*. The recombinant strain was grown in LB medium containing 100 µg ampicillin ml^−1^ and 1 % (w/v) glucose. The expression was induced with 1 mM IPTG at an OD_600_ of 0.6. Cells were harvested 3 h after induction by centrifugation (15 min, 3800 ***g***), resuspended in buffer containing 200 mM Tris/HCl (pH 8.5), 300 mM NaCl and 50 mM KCl, and disrupted by sonication. The debris was removed by centrifugation (15 min, 20 000 ***g***). The glutathione-*S*-transferase (GST)–PPGK fusion was purified by affinity chromatography using Glutathion-Sepharose 4B (GE Healthcare) performed in a batch technique according to the manufacturer’s instructions. To elute All1371 the GST-tagged PPGK was cleaved on the column by PreScission protease (Walker *et al.*, 1994) (GE Healthcare) overnight at 4 °C. The purity of the enzyme was verified by SDS-PAGE.

#### Preparation of cell-free cyanobacterial extract, electrophoresis and protein quantification.

Cyanobacterial cells were collected from liquid cultures (grown with or without nitrogen for 4 or 6 days) by centrifugation (6500 ***g***). Sedimented cells were washed twice with 50 mM Tris/HCl buffer (pH 8.0) and stored at −20 °C. Thawed filaments of *Mastigocladus laminosus* and *F. muscicola* were pretreated by sonication. Cells were disrupted in a swing mill (Retsch MM 301) for 30 min at 30 Hz using glass beads (Ø 0.1 mm). Beads and crude extracts were separated by two sequential centrifugations at 10 000 ***g*** and 4 °C for 10 and 30 min. To remove small molecules the supernatants were purified using DextraSEC PRO2 columns (Applichem). The elution was performed by the original buffer. Protein concentrations were estimated according to Lowry *et al.* (1951) using BSA as reference. SDS-PAGE was performed on slab gels [15 % (w/v) acrylamide, 0.41 % (w/v) methylene-bisacrylamide] (Laemmli, 1970). The gels were stained with Coomassie brilliant blue R250.

#### Determination of molecular mass.

The molecular mass of native All1371 was determined by size exclusion chromatography on a Tricorn Superdex 200 10/300 GL column (GE Healthcare) calibrated with the gel filtration standards purchased from Bio-Rad (γ-globulin, 158 kDa; ovalbumin, 44 kDa; myoglobin, 17 kDa; cytochrome, *ca.* 12.4 kDa). As running buffer 100 mM Tris/HCl (pH 7.5), 200 mM NaCl, 6 mM MgCl_2_ (hereafter basic buffer) and 0.5 mM DTT were used at a flow rate of 0.8 ml min^−1^. Pure All1371 (100 µg) was loaded onto the column. The elution was monitored by measuring *A*_280_. Fractions of 0.5 ml were collected. Aliquots from these fractions were tested for PPGK activity. Precipitated fractions (Bensadoun & Weinstein, 1976) were analysed by SDS-PAGE. Two biological replicates were performed.

#### Activity assays and kinetic analyses.

Glucokinase activity and kinetics of the isolated All1371 were determined *in vitro* by coupling glucose 6-phosphate formation to the glucose-6-phosphate dehydrogenase reaction (Hsieh *et al.*, 1993). Glucose 6-phosphate formation was monitored indirectly by measuring NADH development spectrophotometrically at 340 nm (ϵ_340_ = 6220 M^−1^ cm^−1^). Measurements were done in basic buffer with 0.6 mM NAD, 0.8 mM glucose, 0.01 mM polyP_45_ (phosphate glass type 45; Sigma-Aldrich; hereafter polyphosphate), 5.7 units glucose-6-phosphate dehydrogenase ml^−1^ and 0.26 µg All1371 ml^−1^ at 28 °C. To verify the cation dependency, MgCl_2_ was replaced by MnCl_2_ or water. One unit of PPGK activity was defined as the amount of enzyme which catalyses the formation of 1 µmol glucose 6-phosphate min^−1^. The reaction was started with All1371. All rates were determined from the linear region of the curves. To check the substrate specificity, the following substrates were applied to the assay at final concentrations of 10 µM, 100 µM and 1 mM: polyP_45_, ATP, ADP, AMP, GTP, UTP, CTP and pyrophosphate. Additionally ATP was tested as a substrate at final concentrations of 5 and 10 mM. Polyphosphate was used to check enzyme activity if no activity was detected *in vitro*. To determine the kinetics of All1371, both substrates (glucose, polyphosphate) were varied. The values of *K*_M_ and *k*_cat_ were calculated from the initial rate. Three biological replicates were performed. Kinetic parameters were analysed by Sigma Plot 2006 Enzyme Kinetics Module 1.3 (Systat software). The initial rate was measured for several glucose concentrations at different non-saturating polyP_45_ concentrations. Measurements were also taken for several polyphosphate concentrations at different non-saturating glucose concentrations.

Mannokinase activity was determined by monitoring the formation of NADH spectrophotometrically at 340 nm. Activity was measured in basic buffer including 0.6 mM NAD, 5–70 mM mannose, 0.02 mM polyP_45_, 5.7 units glucose-6-phosphate dehydrogenase ml^−1^, 1.0 units mannose-6-phosphate isomerase ml^−1^, 4.0 units glucose-6-phosphate isomerase ml^−1^ and 23–42 µg All1371 ml^−1^ at 28–30 °C.

When measuring PPGK activities in cell-free extracts, the extracts instead of the pure enzyme were used in standard assays as described above (glucokinase activity). The reaction was started with glucose. To confirm the linearity of the reaction different amounts of the extracts with 23–480 µg protein were added to the reaction mixture. All1371 was applied to the assay as a positive control.

#### Deletion of *all1371* in *Anabaena*.

An *Anabaena* Δ*all1371* mutant was generated by replacing 771 nt including *all1371* [720 nt, genomic region 1625 095–1625 814 (Nakao *et al.*, 2010)] with an antibiotic resistance cassette, not affecting other ORFs.

Upstream and downstream regions of *all1371* were amplified by PCR using genomic *Anabaena* DNA as template. Restriction sites introduced by the primers below are highlighted in the sequence in bold type and termed in parentheses. The upstream region (position 1625 815–1626 817) was amplified using the primers 5′-ATT**GAGCTC**AAGGACGGAAAAAATTACAC-3′ (*Sac*I) and 5′-GAGTATTTACCTTTTT**TCTAGA**GACTGG-3′ (*Xba*I) yielding a PCR product of 973 nt after restriction. The downstream region was amplified using the primer pair 5′-CCCGAAAC**TCTAGA**TGTGACTGGGTATGGGG-3′ (*Xba*I) and 5′-AATG**CTCGAG**AACCAACCTATACCTGTGC-3′ (*Xho*I). The restricted product yielded a 943 nt fragment. The fragments were successively inserted into the pBluescript KSII+ vector (Stratagene) resulting in pKSII+_up_down. The resistance cassette C.K3 containing the neomycin phosphotransferase II gene was received from pRL448 (Elhai & Wolk, 1988a) and inserted into pKSII+_up_down via the *Xba*I site, yielding pKSII+_up-C.K3-down. The C.K3 cassette was inserted in the same direction as *all1371*. The correctness of the sequence was validated by DNA sequencing. To construct pRL271_up-C.K3-down used for deletion, the *Sac*I/*Xho*I fragment excised from the prior plasmid was cloned into plasmid pRL271 (Black *et al.*, 1993). This plasmid was conjugationally transferred to *Anabaena* by triparental mating using *E. coli* strain J53[RP4] and cargo strain *E. coli* HB101[pRL528] (Elhai & Wolk, 1988b). Neomycin-resistant double recombinants were identified by PCR and *sacB* selection (Cai & Wolk, 1990).

#### Viability tests.

Viability tests of *Anabaena* and the Δ*all1371* mutant were carried out as a spot assay on AA-plates (Allen & Arnon, 1955) with or without 10 mM KNO_3_ as a nitrogen source. A 10 µl volume of liquid cultures was applied per spot. These agar plates were exposed to continuous light of 60–70 µmol photons m^−2^ s^−1^ for 6 days. Three biological replicates were tested separately.

#### Generation of a GFP promoter fusion strain.

The *gfp* gene was amplified by PCR using the primer pair 5′-GATGGCTC**TCTAGA**ATGAGTAAAGGAGAAG-3′ and 5′-CT**TCTAGA**TTAATGTTTGTATAGTTCATC-3′ (*Xba*I in bold type, stop codon underlined) and plasmid pJET1.2-GFP as template (Baier, 2013). The *gfp* gene was obtained by *Xba*I digest and inserted in plasmid pKSII+_up_down (see above) via the *Xba*I site, leading to pKSII+_up-gfp-down. By this means, the *gfp* gene was integrated in this plasmid into the upstream region of *all1371* 32 nt after the transcription start site. This plasmid was used as template in a PCR performed with oligonucleotides 5′-CTATAGGGC**GAATTC**GAGCTCAAGGACGG-3′ and 5′-GTGTTCTTCTCC**GAATTC**CCATAC-3′ (*Eco*RI sites in bold type). Finally, the PCR product was inserted into pRL1049 (Black & Wolk, 1994) via the *Eco*RI sites, resulting in vector pRL1049-up-gfp-down, which was validated by DNA sequencing. To generate *Anabaena all1371* GFP promoter fusion strains, pRL1049-up-gfp-down_*all1371* was introduced in the *Anabaena* wild-type and Δ*all1371*. Conjugation was performed as described above. Positive exconjugants selected on AA-agar plates (Allen & Arnon, 1955) containing 4 µg spectinomycin ml^−1^ and 1 µg streptomycin ml^−1^ were checked by PCR using primers 5′-GCCTGCATTTGGTGCTGGACTGG-3′ and 5′-GGTCTGCTAGTTGAACGCTTCC-3′. The plasmid pRL1049-up-gfp-down_*all1371* was self-replicating in these exconjugants.

#### Confocal microscopy.

For confocal microscopy *Anabaena* and mutant strains (Δ*all1371*, promoter fusion) were grown as liquid cultures with and without nitrate for 4 days. Fluorescence in cells of the *Anabaena* Δ*all1371* promoter fusion strain was visualized with a laser-scanning confocal microscope (Olympus FV-1000MPE). GFP was excited by an argon ion laser (488 nm irradiation). Fluorescence emission was recorded at 500–545 nm (for GFP) and 570–670 nm (for chlorophyll fluorescence) using a 60× water-immersion objective (Olympus IX-81 60×/1.2 Water UPlanSApo, DIC, fourfold zoom). All confocal images for each experiment were acquired using identical adjustments. The GFP fluorescence was quantified using Olympus Fluoview version 3.1. The fluorescence of a heterocyst was compared with that of the two adjacent vegetative cells. The Δ*all1371* mutant strain without GFP was used as control. Background fluorescence was subtracted.

## Results

### All1371 as putative PPGK

**Fig. 1.**
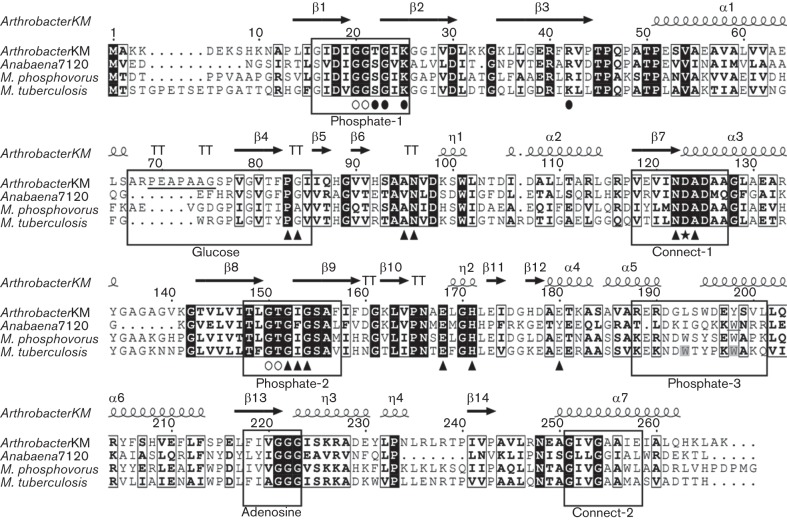
Primary structural alignments of different PPGKs. Aligned primary structures from *Arthrobacter* sp. strain KM, *Microlunatus phosphovorus* (Tanaka *et al.*, 2003), *Mycobacterium tuberculosis* (Hsieh *et al.*, 1993; Phillips *et al.*, 1999) and *Anabaena* sp. PCC 7120. Strictly conserved residues are shaded in black; similar residues are framed in black. Putative structural and functional domains are enclosed in boxes. Secondary structural elements [e.g. α helices, β sheets, turn-turns (TT)] of the polyP/ATP-glucomannokinase from *Arthrobacter* sp. strain KM are depicted above the alignment. Residues associated with β-d-glucose binding are marked with filled triangles. The heptapeptide is underlined. The catalytic aspartate (D) is highlighted with a star. Residues involved in the binding of both phosphate molecules used as ligands (open, phosphate A; filled, phosphate B) are marked with ovals (Mukai *et al.*, 2004).

The ORF *all1371* from *Anabaena* was assumed to encode a putative PPGK (EC 2.7.1.63), as its amino acid sequence has sequence similarity to several well-characterized bacterial PPGKs, including the polyphosphate/ATP-glucomannokinase from *Arthrobacter* sp. strain KM (Mukai *et al.*, 2003, 2004) (53.1 % similarity), the polyphosphate-dependent PPGK from *Microlunatus phosphovorus* (Tanaka *et al.*, 2003) (46.0 % similarity) and the polyphosphate/ATP-dependent glucokinase from *Mycobacterium tuberculosis* (Hsieh *et al.*, 1993, 1996a, b) (47.4 % similarity). Comparison of these sequences, including secondary structures extrapolated from the crystal structure of the polyphosphate/ATP-glucomannokinase of *Arthrobacter* sp. strain KM (1WOQ) (Mukai *et al.*, 2003, 2004), revealed the presence of some highly conserved common motifs (Fig. 1). Seven structural and functional motifs (Fig. 1, boxed) were found in all of the sequences including All1371.

### Purification of All1371 and molecular mass determination

**Fig. 2.**
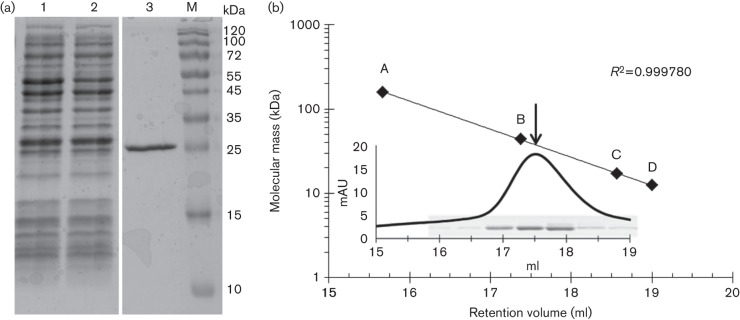
Purification of recombinant All1371 and gel filtration. (a) SDS-PAGE (15 %) analysis of samples from the various purification steps performed after affinity chromatography. Lane 1, cell-free extract of recombinant *E. coli*, 15 µg protein; lane 2, flow-through, 15 µg protein; lane 3, All1371 after elution, 2 µg; lane M, protein standard. (b) All1371 was subjected to gel filtration calibrated with protein standards (filled diamonds; see Methods for details). The elution profile (indicating the elution position of All1371, arrow) and SDS-PAGE results of the obtained fractions are shown in the inset.

The N-terminal GST-fusion protein of All1371 was expressed for 3 h in *E. coli* BL21(DE3) carrying pGEX_*all1371*. After on-column cleavage with the PreScission protease, the 246 aa enzyme was eluted and analysed by SDS-PAGE (Fig. 2a). The enzyme appeared as a single band of 26 kDa (Fig. 2a, lane 3; apparent molecular mass). To investigate the oligomeric state of All1371, the recombinant PPGK was analysed by size-exclusion chromatography. A single symmetrical peak (Fig. 2b, inset) of approximately 39.0 kDa was obtained. Fractions corresponding to the protein elution peak showed a single protein band of 26 kDa on SDS-PAGE (Fig. 2b, inset), and exhibited polyphosphate-dependent activity *in vitro* (data not shown). The biochemical and kinetic properties of All1371 are summarized in Table 1.

**Table 1.** Biochemical and kinetic properties of All1371 Measurements were performed at 28 °C, pH 7.5; *n*≥3; 100 % = 107.1 U mg^−1^.

Property		Value (mean±sd)
Molecular mass (kDa)	Native complex*	39
	Monomer, apparent†	26.0
	Monomer, calculated‡	26.6
Oligomeric structure		Monomer or homodimer
*K*_M_ (mM)	Glucose	0.118±0.01
*K*_M_ (µM)	Polyphosphate	1.76±0.26
*v*_max_ (U mg^−1^)	Glucose, polyphosphate	107.1±15.3
*k*_cat_ (s^−1^)	Glucose, polyphosphate	48.2±6.9
*K*_0.5_ (mM)	Mannose	24.3±2.36
*v*_max_ (U mg^−1^)	Mannose	0.43±0.04
*k*_cat_ (s^−1^)	Mannose	0.21±0.017
Phosphoryl donor specificity (% *v*_max_)	Polyphosphate	100
	ATP, GTP, UTP, CTP, ADP, AMP	0
	Pyrophosphate	0
	Without polyphosphate	0

* Determined by size exclusion chromatography.

† Determined by SDS-PAGE.

‡ Calculated according to the primary structure, including the linker peptide of the GST tag.

### Biochemical properties of All1371

Our enzyme activity assays indicated that purified All1371 uses polyphosphate to phosphorylate glucose and mannose, with a higher preference for glucose (Table 1). All1371 activity was strictly dependent on the presence of Mg^2+^ or Mn^2+^ (data not shown). All1371 had high substrate specificity and acted as a strict polyphosphate-dependent enzyme. No other phosphoryl group donor was accepted. Kinetic analysis indicated that the reactions of All1371 with polyphosphate and glucose followed Michaelis–Menten kinetics. The *K*_M_ values for polyphosphate and glucose were 1.76 µM and 0.118 mM, respectively (at 28 °C and pH 7.5). The maximum rate of All1371-mediated catalysis was 107 U mg ^−1^, yielding a *k*_cat_ of 48.2 s ^−1^ (Table 1). Furthermore, our kinetic analysis revealed that All1371 had a *k*_cat_ of 0.19 s^−1^ and a *K*_0.5_ of 24.3 mM for mannose (Table 1).

**Fig. 3.**
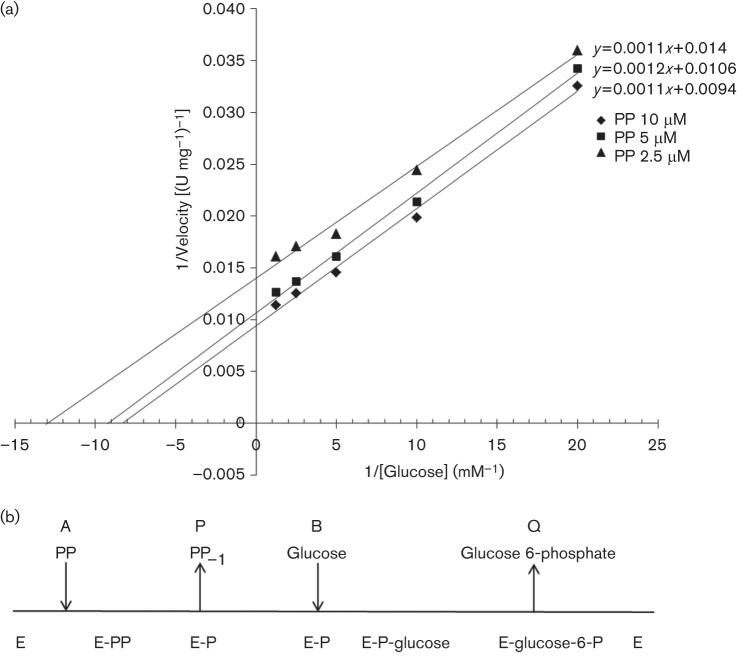
Kinetics of the All1371 reaction. (a) Activity of All1371. Primary double-reciprocal plot of initial velocity with glucose as the variable substrate and different concentrations of polyP_45_ (PP) as the fixed substrate (pH 7.5; *n* = 3). (b) Schematic of the bi-bi ping-pong mechanism. Polyphosphate (PP) acts as the first substrate (A) by covalently binding to PPGK (E). The first product (P), polyphosphate reduced at one phosphate (PP_–1_), is released, and binding of the second substrate, glucose (B), occurs on the phosphorylated enzyme (E-P). Finally, the second product, glucose 6-phosphate (Q), is released and the enzyme is restored (E).

To characterize the enzymic mechanism of All1371, additional kinetic analyses with glucose and polyphosphate were performed. The initial rate of All1371 activity was determined with varying concentrations of glucose and fixed concentrations of polyphosphate. We obtained a linear double reciprocal plot with parallel lines (Fig. 3a). When polyphosphate was varied, we obtained a similar graph with parallel lines (data not shown).

### Distribution of putative PPGKs in cyanobacteria and PPGK activity in cell-free cyanobacterial extracts

To examine the distribution of PPGKs among cyanobacteria, we performed a blastp search (Altschul *et al.*, 1997) against all sequenced cyanobacterial genomes (September 2013; 141 genomes) using the amino acid sequence of All1371 as the query. Our analysis revealed that in 34 % of all sequenced cyanobacteria a putative PPGK is present. PPGKs were found in all five sections of cyanobacteria (Table 2) with the highest frequency in the heterocyst-forming species of Section IV (85 %) and Section V (54.5 %), followed by the non-heterocystous species of Section III (52.9 %), Section II (50 %) and Section I (5.7 %). More complete data are presented in Table S1 (available in the online Supplementary Material). Cell-free extracts of some of these cyanobacteria were tested for specific PPGK activity under different nitrogen conditions, including *Anabaena* (Section V), *Mastigocladus laminosus* (Section V) (Nürnberg *et al.*, 2014), *F. muscicola* (Section V) and *Synechocystis* (Section I), the last lacking a predicted PPGK (negative control). As expected, cell-free extracts of *Synechocystis* did not show any PPGK activity, whereas those of *Anabaena*, *Mastigocladus laminosus* and *F. muscicola* exhibited detectable PPGK activity (Table 3). In *Anabaena*, PPGK activity was increased slightly under nitrogen depletion. PPGK activity in cell-free extracts from the two diazotrophic, branched filamentous cyanobacterial strains of Section V was three- to fourfold higher than in non-branched *Anabaena* cells (Table 3). Interestingly, we found a decrease of PPGK activity in extracts of Section V cells grown without combined nitrogen (Table 3).

**Table 2.** Distribution of PPGKs among cyanobacteria

	Section				
	I	II	III	IV	V
No. of PPGKs	4	3	18	17	6
No. of cyanobacteria	70	6	34	20	11
Percentage	5.7	50.0	52.9	85.0	54.5

**Table 3.** PPGK activities in cell-free extracts of cyanobacteria Measurements were performed at 28–30 °C and pH 7.5; *n*≥3; nd, not detectable (≤1 nmol min^−1^ mg^−1^).

Strain	Section	Growth conditions	PPGK activity (nmol min^−1^ mg^−1^) (mean±sd)
*Anabaena*	IV	+N	4.3±1.3
		−N	5.8±1.3
*Anabaena* Δ*all1371*	IV	+N	nd
		−N	nd
*F. muscicola*	V	+N	15.3±2.3
		−N	13.1±2.1
*Mastigocladus laminosus*	V	+N	23.6±0.7
		−N	13.9±3.2
*Synechocystis*	I	+N	nd
		−N	nd

### Viability of Δ*all1371*

**Fig. 4.**
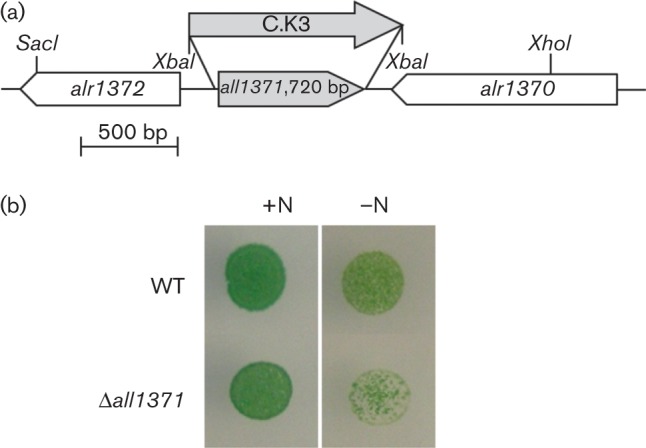
The Δ*all1371* mutant. (a) Schematic of the chromosomal region surrounding *all1371* and the gene inactivation strategy, in which *all1371* was replaced with the C.K3 cassette. (b) Viability analyses of *Anabaena* wild-type (WT) and the *all1371* knockout mutant strain (Δ*all1371*) on AA-agar plates containing 10 mM KNO_3_ (+N) or lacking combined nitrogen (–N). In total, 4.8 ng chlorophyll *a* per spot was plated. The plates were incubated under continuous light for 6 days.

To confirm a function of All1371 *in vivo*, we generated a mutant strain in which ORF *all1371* was replaced with the neomycin-resistance cassette, C.K3 (Fig. 4a). Complete segregation of the mutant was validated by PCR (Fig. S1), and no PPGK activity was detected in cell-free extracts of Δ*all1371* (Table 3). Using light and fluorescence microscopy, we monitored the shape and autofluorescence of cells. When the Δ*all1371* mutant was deprived of combined nitrogen, the filaments showed both vegetative cells and morphologically mature heterocysts. Analysis of Δ*all1371* mutant and WT cells under different light and nitrogen conditions revealed that viability of the mutant was distinctly decreased under combined nitrogen-limiting conditions (Fig. 4b, Fig. S2). This effect was increased under light–dark cycle conditions, but in this case we also noted a reduced viability of the mutant in the presence of nitrate (Fig. S3b).

### Expression analysis using a GFP promoter fusion

**Fig. 5.**
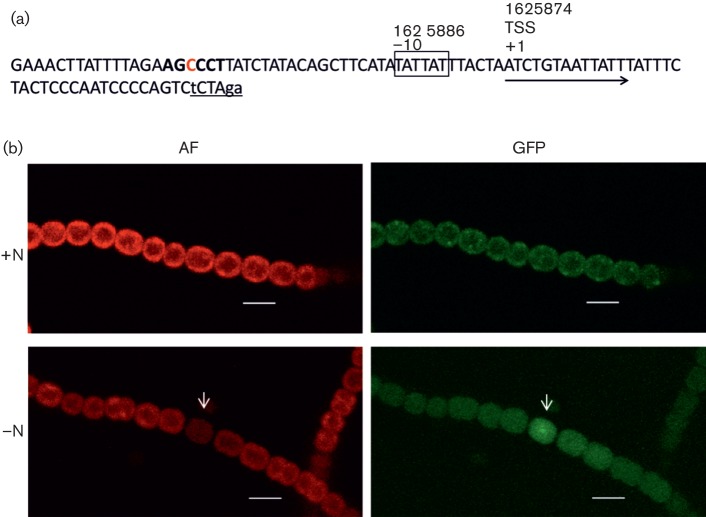
*All1371* promoter activity in heterocysts. (a) The promoter region of *all1371*, including the TSS and the −10 region (boxed) (Mitschke *et al.*, 2011), was integrated into a self-replicating plasmid. A promoter-less *gfp* gene was integrated via the underlined *Xba*I site (altered bases are depicted in lower case); the presumed DIF+ domain (AGCCCT) is shown in bold. (b) Fluorescence in the *Anabaena* Δ*all1371* promoter fusion strain grown under N_2_-fixing conditions (–N) and grown with combined nitrogen (+N). A heterocyst is indicated with an arrow. Bars, 5 µm. AF, auto-fluorescence (red); GFP, GFP fluorescence (green).

The transcriptional changes experienced by *Anabaena* during nitrogen-depletion-induced cell differentiation were recently analysed by Mitschke *et al.* (2011). They identified numerous transcription start sites (TSSs) in *Anabaena*, including a TSS of *all1371* at position 1625 874. The organization of the predicted *all1371* promoter region is depicted in Fig. 5(a). We identified a putative palindromic motif that is likely to be a (DIF)+ motif (Mitschke *et al.*, 2011). This putative (DIF)+ motif displays an inverse orientation and one mismatch [Fig. 5a; (DIF)+ in bold type, mismatch in red, AGCCCT].

To investigate the expression of *all1371* along the filaments, the *gfp* gene was transcriptionally fused to the *all1371* promoter and transformed into the Δ*all1371* mutant strain. In the absence of combined nitrogen, we noted distinct GFP fluorescence in the mature heterocysts of 4-day-old filaments of the promoter fusion strain (Fig. 5b). An overview of fluorescence (GFP, autofluorescence) of the promoter fusion strain is given in Fig. S4. No distinct GFP fluorescence was observed in the filaments of the promoter fusion strain when combined nitrogen was supplied (Fig. 5b) compared with Δ*all1371* (Fig. S5). GFP fluorescence in heterocysts was first detected 24 h after nitrogen step down, and persisted until the filaments were harvested 4 days later (data not shown). Quantification of GFP fluorescence of numerous cells in the promoter–*gfp* fusion strain in comparison with cells of the parent strain Δ*all1371* confirmed our microscopic observations: the fluorescence intensity in heterocysts (*n* = 42) of the promoter fusion strain was ~2.1-fold higher than in vegetative cells (*n* = 71). The ratio of fluorescence intensity from heterocysts (*n* = 18) to vegetative cells (*n* = 34) in filaments of the parent strain Δ*all1371* was 1 : 0.9 (Table S2).

## Discussion

### Identification of the putative PPGK, All1371

The structural and functional motifs found in all the aligned amino acid sequences (Fig. 1, boxed) are predicted to interact with the substrates glucose, mannose, ATP and polyphosphate (Liao *et al*., 2012; Mukai *et al.*, 2003). Five of the identified domains have been proposed to interact with the ATP molecule: phosphate-1 and phosphate-2 are involved in binding β-phosphates and γ-phosphates, while connect-1, connect-2 and the adenosine motif interact with the adenine ring of ATP (Mukai *et al.*, 2003). The conserved aspartate residue in the connect-1 region (Fig. 1, star) is believed to be essential for catalytic activity (Arora *et al.*, 1991; Mukai *et al.*, 2004). The glucose motif has been suggested to participate in glucose binding. The heptapeptide PEAPAAG (Fig. 1, underlined) was proposed to be responsible for mannose phosphorylation in the sequence of *Arthrobacter* sp. strain KM (Mukai *et al.*, 2003). The phosphate-3 motif was predicted to be a binding region for polyphosphate (Liao *et al*., 2012). Residues Trp193 and Trp198 (Fig. 1, grey shading) were proposed to be essential for catalytic activity in *Mycobacterium tuberculosis* (Phillips *et al.*, 1999). A residue equivalent to Trp198 is present in the amino acid sequence of *Anabaena* (Fig. 1, underlined W). Furthermore, the phosphate-1 domain is likely to contain a putative polyphosphate-binding site, as both the anionic phosphates used as ligands in a crystallographic study (Mukai *et al.*, 2004) bound at highly conserved amino acid residues similar to Lys25. It was proposed that there may be shared ATP- and polyphosphate-binding sites in the phosphate-1 and phosphate-2 regions (Mukai *et al.*, 2004). Thus, the present amino acid sequence analysis of All1371 (Fig. 1) and the previous findings in similar proteins collectively suggest that All1371 functions as a PPGK.

### Purification of All1371, and molecular mass determination 

The purified All1371 appeared as a single protein band of 26 kDa in SDS-PAGE analysis (Fig. 2a, lane 3). This result is consistent with the expected molecular mass of 26.6 kDa calculated with the ProtParam tool (http://web.expasy.org/protparam/) (Wilkins *et al.*, 1999) for one monomer of the recombinant All1371.

The protein peak of 39 kDa obtained in size-exclusion chromatography (Fig. 2b) indicates that the native enzyme may exist as either a monomer or a homodimer. PPGK homodimers have also been reported in *Mycobacterium tuberculosis*, *Propionibacterium shermanii* and *Propionibacterium arabinosum* (Phillips *et al.*, 1999), whereas the polyphosphate/ATP-dependent glucomannokinase of *Arthrobacter* sp. strain KM was determined to exist as a monomer (Mukai *et al.*, 2003).

### Biochemical properties of All1371

All1371 uses polyphosphate exclusively to phosphorylate glucose and mannose (Table 1) and is strictly dependent on the presence of divalent cations. This requirement for divalent cations is shared with the PPGKs of *Microlunatus phosphovorus* (Tanaka *et al.*, 2003), *Arthrobacter* sp. (Mukai *et al.*, 2003), *Mycobacterium tuberculosis* and *Mycobacterium phlei* (Szymona & Ostrowski, 1964; Szymona & Widomski, 1974). Recently, Mg^2+^ was found to be an indispensable cofactor for the PPGK of *Thermobifida fusca* (Liao *et al*., 2012). Here, we report that All1371 showed high substrate specificity and acted as a strict polyphosphate-dependent enzyme. This result is a notable feature, as most of the previously described glucokinases utilized either ATP alone, or ATP and polyphosphate. The previous *in vitro* studies on PPGKs revealed that these enzymes were often bi-functional and not restricted to polyphosphate. For example, the PPGKs from *Mycobacterium tuberculosis* (Hsieh *et al.*, 1996a), *Propionibacterium shermanii* (Phillips *et al.*, 1993) and *Corynebacterium glutamicum* (Lindner *et al.*, 2010) were also able to use ATP or GTP. The present work showed that, along with the PPGK of *Microlunatus phosphovorus* (Tanaka *et al.*, 2003), All1371 is one of only two known PPGKs that uses only polyphosphate as its phosphate donor.

Kinetic analyses of All1371 (Table 1) revealed a relatively low *K*_M_ value obtained for polyphosphate (1.76 µM), suggesting that All1371 has a high affinity for its sole substrate. In comparison with the *K*_M_ values for polyphosphate and glucose (Table 1), the polyphosphate- and ATP-dependent PPGK from *Corynebacterium glutamicum* yielded *K*_M_ values of 0.2 mM for polyP_45_ and 1 mM for glucose (Lindner *et al.*, 2010); the PPGK of *Propionibacterium shermanii* yielded a *K*_M_ value of 1.2 µM for polyP_35_ (Phillips *et al.*, 1993); the PPGK of *Mycobacterium tuberculosis* yielded a *K*_M_ value of 4.6 µM for polyP_35_ (Phillips *et al.*, 1999); and the PPGK of *Microlunatus phosphovorus* yielded a *K*_M_ of 3.8 mM for polyP_30_ (Tanaka *et al.*, 2003). The turnover number of All1371 of 48.2 s ^−1^ (Table 1) is comparable with the *k*_cat_ value of 57.0 s^−1^ determined for the PPGK from *Propionibacterium shermanii* against polyP_35_ (Phillips *et al.*, 1999).

According to our analyses, All1371 is a polyphosphate-dependent glucomannokinase. Interestingly, the heptapeptide in the sequence of *Arthrobacter* sp. strain KM (Fig. 1, underlined), which is assumed to be responsible for mannose phosphorylation (Mukai *et al.*, 2004), is not present in the corresponding *Anabaena* sequence. The results of additional kinetic analyses (Fig. 3a) were consistent with the so-called ‘bi-bi ping-pong’ mechanism (Cleland, 1963). As illustrated in Fig. 3(b), this mechanism is a particular multi-substrate reaction that includes two substrates and two products (bi-bi) and is characterized by alternating processes of substrate binding and product release (ping-pong) for the two substrates. In a first step, polyphosphate is covalently bound to All1371, which is then phosphorylated. Approximately one orthophosphate-reduced polyphosphate is released from the enzyme as the first product. In a second step, glucose is bound to the phosphorylated enzyme, and the second substrate is phosphorylated. Glucose 6-phosphate is released as a second product, and the enzyme returns to its initial state (Fig. 3b). In contrast, the ATP/polyphosphate-dependent PPGK of *Mycobacterium tuberculosis* (Hsieh *et al.*, 1996a) and the ATP-dependent glucokinase from *Streptomyces coelicolor* (Imriskova *et al.*, 2005) were both found to display ordered bi-bi sequential mechanisms. The ordered bi-bi sequential mechanism differs from the bi-bi ping-pong mechanism in that both substrates (glucose and ATP or polyphosphate) bind to the enzyme first before the two products are released.

### Putative PPGKs in cyanobacteria

Cyanobacteria may be grouped into five sections according to their morphology (Rippka *et al.*, 1979). While species of Sections I and II are unicellular forms, those of Sections III, IV and V show filamentous forms. Cyanobacteria of Sections IV and V are additionally able to form heterocysts. The highest level of complexity is seen among Section V strains, which form true branches within their filaments (Golubic *et al.*, 1996). Diazotrophic growth has been observed in both unicellular and filamentous strains (reviewed by Stal, 1995). Our blastp search revealed that PPGKs were found very frequently in cyanobacteria of Sections IV and V, which are all diazotrophic strains forming heterocysts. All genomes of the analysed Section IV and Section V strains contained PPGK genes. In about half of the analysed genomes of Section III we found putative PPGK genes (52.9 %). In 11 of these 18 PPGK gene-containing genomes (61 %) we also found nifH genes encoding the key enzyme of N_2_ fixation (Table S1). Some of these Section III organisms are known to fix N_2_ under micro-oxic conditions, such as *Pseudanabaena* sp. ATCC 27183 (Rippka & Waterbury, 1977) (synonymous with *Pseudanabaena* sp. PCC 6802). Among cyanobacteria of Section II, 50 % of sequenced unicellular strains were also predicted to contain a PPGK. All are known to fix N_2_ under anaerobic conditions (Rippka *et al.*, 1979) or to have a nitrogenase complex (Rippka & Waterbury, 1977), or a putative dinitrogenase has been annotated in the genome (Markowitz *et al.*, 2012). Furthermore, strains belonging to *Chroococcidiopsis* are closely related to the heterocyst-forming cyanobacteria (Fewer *et al.*, 2002). These facts may suggest a possible correlation between PPGK appearance and the ability to fix N_2_ under anoxic/micro-oxic conditions provided by either heterocysts or the environment. This presumption is supported by the results obtained by analysing Section I organisms. Only four (5.7 %) of the unicellular strains of Section I were found to contain a putative PPGK. *Synechococcus* sp. PCC 7335 and *Synechococcus* sp. PCC 7502 arose through morphological transition events (Robertson *et al.*, 2001; Shih *et al.*, 2013). Interestingly, putative PPGKs were not found in the genomes of *Cyanothece* strains that are able to grow diazotrophically in diurnal rhythm. Based on the present findings, we hypothesized that the presence of a PPGK in cyanobacterial genomes is strongly related to the organism’s ability to fix N_2_ in heterocysts. A correlation between PPGK appearance and an organism’s ability to fix N_2_ under anoxic conditions is possible but has to be analysed further, especially from a phylogenetic point of view.

To determine whether PPGK activity was present *in vivo*, PPGK activity in cell-free extracts of some heterocyst-forming cyanobacteria with putative PPGKs was determined. As summarized in Table 3, PPGK activity in *Anabaena* is increased after 4 days of nitrogen depletion. An increase of PPGK activity under this condition is in line with the results of Flaherty *et al*. (2011). Using deep sequencing analyses performed 21 h after nitrogen deprivation, they found a 4.8-fold increase in the mRNA expression level of *all1371*. Furthermore, the increased PPGK activity is in line with a previous report (Thompson *et al.*, 1994), showing that in *Anabaena flos-aquae*, phosphate is stored as sugar phosphate under N_2_-fixing conditions, but as polyphosphate in the presence of combined nitrogen. We found that PPGK activity was higher in cell-free extracts from thermophilic Section V strains of *F. muscicola* and *Mastigocladus laminosus* than in *Anabaena* (Table 3). A higher *in vitro* activity might be the result of a higher robustness of the PPGK due to its thermophilic origin (Beadle *et al.*, 1999). Interestingly, we observed an increased PPGK activity in cell-free extracts obtained from these strains grown in the presence of nitrate than under diazotrophic conditions. The higher complexity of Section V strains differing in the regulation of diazotrophic growth (Nürnberg *et al.*, 2014) might explain this observation.

### Viability of Δ*all1371*

The impaired viability of the mutant implies that All1371 plays an important role in providing glucose-6-phosphate in *Anabaena*, supporting the canonical hexokinase. This is supported by the PPGK activity measured in cell-free extracts of *Anabaena* obtained from nitrate-supplemented cultures (Table 3). Under diazotrophic conditions heterocysts are not able to fix carbon dioxide. Carbon compounds, probably in the form of sucrose (Curatti *et al.*, 2002), are imported from vegetative cells. NAD(P)H, needed as a reducing equivalent, is generated in heterocysts (Maldener & Muro-Pastor, 2010). There, glucose 6-phosphate is used as substrate for glucose-6-phosphate dehydrogenase, a main enzyme of the oxidative pentose phosphate pathway. Because of the decreased viability of the mutant observed under diazotrophic conditions, we conclude that All1371 may represent an alternative enzyme completing the hexokinase under ATP-consuming (diazotrophic) growth conditions.

### Expression analysis using a GFP promoter fusion

In cyanobacteria, the nitrogen-regulated genes are mainly controlled by the transcriptional regulators NtcA and HetR (Kumar *et al.*, 2010). Recently, chromatin immunoprecipitation analysis followed by high-throughput sequencing was used to identify all of the NtcA-binding sites of *Anabaena* at 3 h after a nitrogen step down (Picossi *et al.*, 2014). Interestingly, they detected an internal NtcA-binding site in *all1371* whereas the impact of binding of NtcA remains unclear. Further HetR-controlled promoters characterized by an differentiation-related change (DIF)+ motif (TCCGGA, a palindrome at or close to position −35) were identified by comparing results found in *Anabaena* with a Δ*hetR* mutant 8 h after a nitrogen step down (Mitschke *et al.*, 2011). The putative (DIF)+ motif with an inverse orientation located in the promoter region of *all1371* (Fig. 5 a) additionally indicates that the promoter might be HetR-dependent. In fact, the promoter of *all1371* responded to nitrogen depletion in WT but not in the Δ*hetR* mutant, indicating a HetR dependency (W. Hess, personal communication). Our results obtained with a GFP promoter fusion strain show that the *all1371* promoter activity is particularly enhanced under nitrogen starvation in mature heterocysts. N_2_ fixation in heterocysts is an energy-intensive process requiring 16 molecules of ATP to reduce one molecule of N_2_ (Hill *et al.*, 1981; Howard & Rees, 1996). The ability of PPGKs to utilize polyphosphate instead of ATP for glucose phosphorylation might allow the heterocysts to save ATP for the essential process of N_2_ fixation.
